# Aero-ZnS prepared by physical vapor transport on three-dimensional networks of sacrificial ZnO microtetrapods

**DOI:** 10.3762/bjnano.15.44

**Published:** 2024-05-02

**Authors:** Veaceslav Ursaki, Tudor Braniste, Victor Zalamai, Emil Rusu, Vladimir Ciobanu, Vadim Morari, Daniel Podgornii, Pier Carlo Ricci, Rainer Adelung, Ion Tiginyanu

**Affiliations:** 1 National Center for Materials Study and Testing, Technical University of Moldova, Chisinau, Republic of Moldova; 2 Academy of Sciences of Moldova, Chisinau, Republic of Moldova; 3 Institute of Electronic Engineering and Nanotechnology „D. Ghitu”, Technical University of Moldova, Chisinau, Republic of Moldova; 4 Institute of Applied Physics, State University of Moldova, Chisinau, Republic of Moldova; 5 Department of Physics, University of Cagliari, Italy; 6 Department of Material Science, Kiel University, Kiel, Germany

**Keywords:** aeromaterial, crystallographic structure, luminescence, physical vapor transport, scanning electron microscopy (SEM), X-ray diffraction (XRD)

## Abstract

Aeromaterials represent a class of increasingly attractive materials for various applications. Among them, aero-ZnS has been produced by hydride vapor phase epitaxy on sacrificial ZnO templates consisting of networks of microtetrapods and has been proposed for microfluidic applications. In this paper, a cost-effective technological approach is proposed for the fabrication of aero-ZnS by using physical vapor transport with Sn_2_S_3_ crystals and networks of ZnO microtetrapods as precursors. The morphology of the produced material is investigated by scanning electron microscopy (SEM), while its crystalline and optical qualities are assessed by X-ray diffraction (XRD) analysis and photoluminescence (PL) spectroscopy, respectively. We demonstrate possibilities for controlling the composition and the crystallographic phase content of the prepared aerogels by the duration of the technological procedure. A scheme of deep energy levels and electronic transitions in the ZnS skeleton of the aeromaterial was deduced from the PL analysis, suggesting that the produced aerogel is a potential candidate for photocatalytic and sensor applications.

## Introduction

Porous materials represent a class of solid-state networks widely used in adsorptive and photocatalytic removal of pollutants from the atmosphere and from water, in other catalytic processes, including photocatalytic water splitting, in energy production and storage, in microfluidic systems, in drug delivery and other biomedical applications, in sensing, in electronic, photoelectronic, optoelectronic and nanophotonic devices, and in other specific applications, such as electromagnetic interference shielding and microwave absorbing materials. Among inorganic porous materials, several groups predominate, such as metal halide perovskites (MHP) [1], Si and III–V semiconductors [2–6], chalcogenides [7–9], and metal oxides [1,9–10]. Metal oxides include TiO_2_, ZnO, Al_2_O_3_, WO_3_, Cu_2_O, CuO, SnO_2_, Fe_2_O_3_, Bi_2_O_3_, Ag_3_PO_4_, BiWO_4_, BiVO_4_, BiFeO_3_, and SeTiO_3_, while chalcogenides are represented by ZnS, ZnSe, CdS, PbS, CdSe, SnS_2_, and Bi_2_S_3_.

Among porous semiconductor materials, recently developed super-lightweight ones with ultrahigh degree of porosity, the so-called aeromaterials, are of special interest [11–18]. Aeromaterials are similar to aerogels, which are widely explored and used in various applications. Aerogels include inorganic [19–24], organic [21–25], and hybrid composite [26–27] materials.

Aeromaterials have been prepared on the basis of sacrificial nano/microstructured templates. Nanofibrillated cellulose has been used as a sacrificial template for the preparation of inorganic nanotube networks, such as titanium dioxide, zinc oxide, and aluminum oxide nanotube networks, by atomic layer deposition [20]. Another aeromaterial, so called aerographite, has been produced by a one-step chemical vapor deposition process with a simultaneous and complete removal of the template material consisting of highly porous 3D networks built from interconnected micrometer-thick ZnO rods with the shape of tetrapods [28]. This aerographite material is a tubular graphitic carbon mimicry of a sacrificial ZnO template architecture in which ZnO has been replaced by carbon from the toluene precursor.

Sacrificial porous ZnO networks of microtetrapods have also been used for the preparation of the abovementioned semiconductor-based aeromaterials. Most of these aeromaterials have been produced by hydride vapor phase epitaxy (HVPE) [11–18]. Particularly, an aero-ZnS material exhibiting hydrophilic properties under tension and hydrophobic properties when compressed against water was fabricated using HVPE of CdS on sacrificial ZnO microtetrapods through the simultaneous or subsequent transformation of CdS into ZnS and the removal of the sacrificial ZnO crystals [16]. Self-propelled liquid marbles have been demonstrated on the basis of this aeromaterial. However, HVPE is an expensive technology.

The goal of this paper is to demonstrate a cost-effective vapor transport approach for the preparation of ZnS aeromaterials.

## Results and Discussion

**Figure 1:**
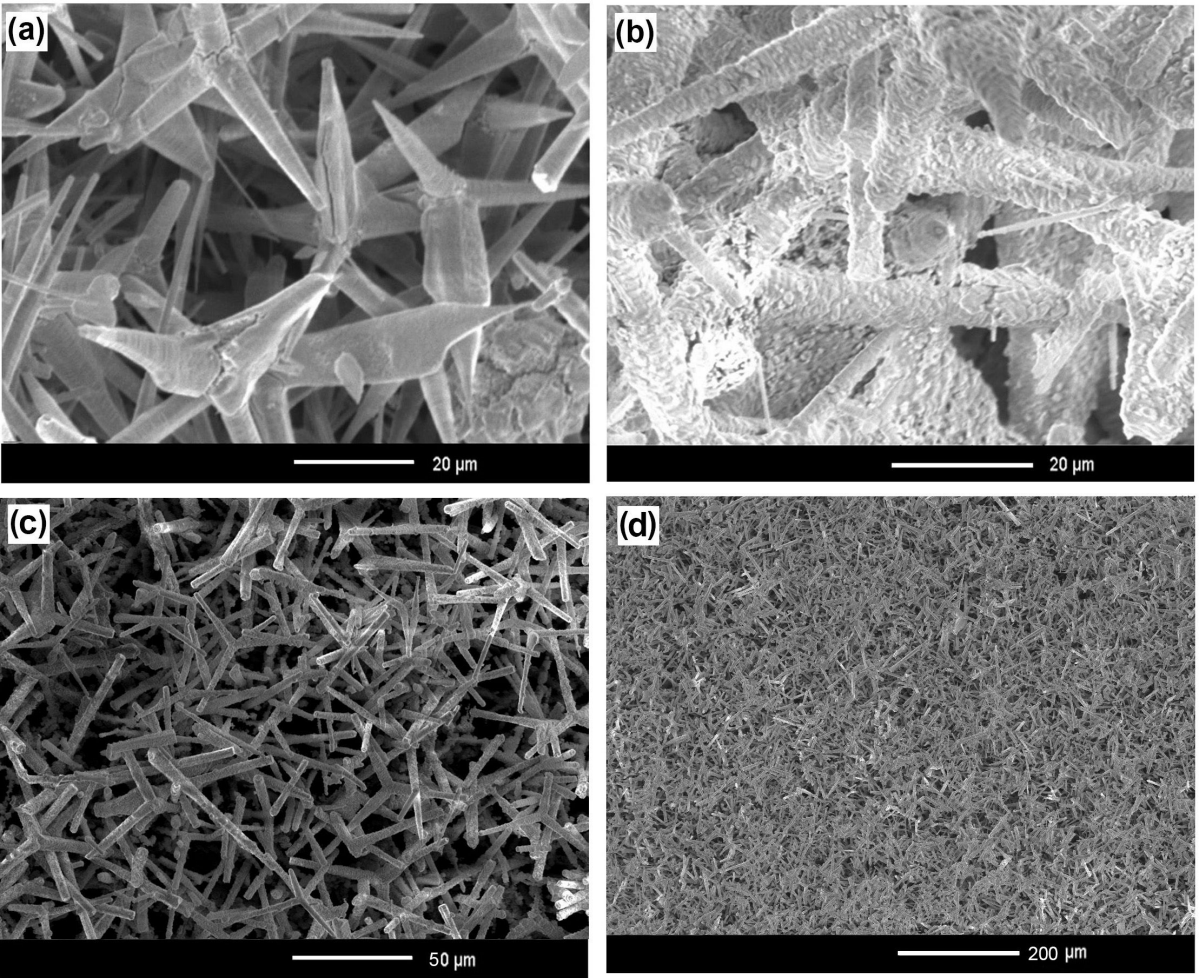
SEM images of ZnS microtetrapods obtained from ZnO microtetrapods after a technological procedure carried out for (a) 4 h and (b) 8 h. (c, d) SEM images taken at low magnification.

The initial ZnO template, consisting of microtetrapods with microrod arms of 10–30 µm length, was transformed, as a result of a technological procedure (see Experimental section) carried out over a period of 4 h, to ZnS microtetrapods with hollow arms, which are similar to those previously produced by HVPE [16], as shown in Figure 1a. When the procedure took 8 h, the geometry of the microtetrapods was preserved; however, the surface of their arms became more granulated, as shown in Figure 1b.

**Figure 2:**
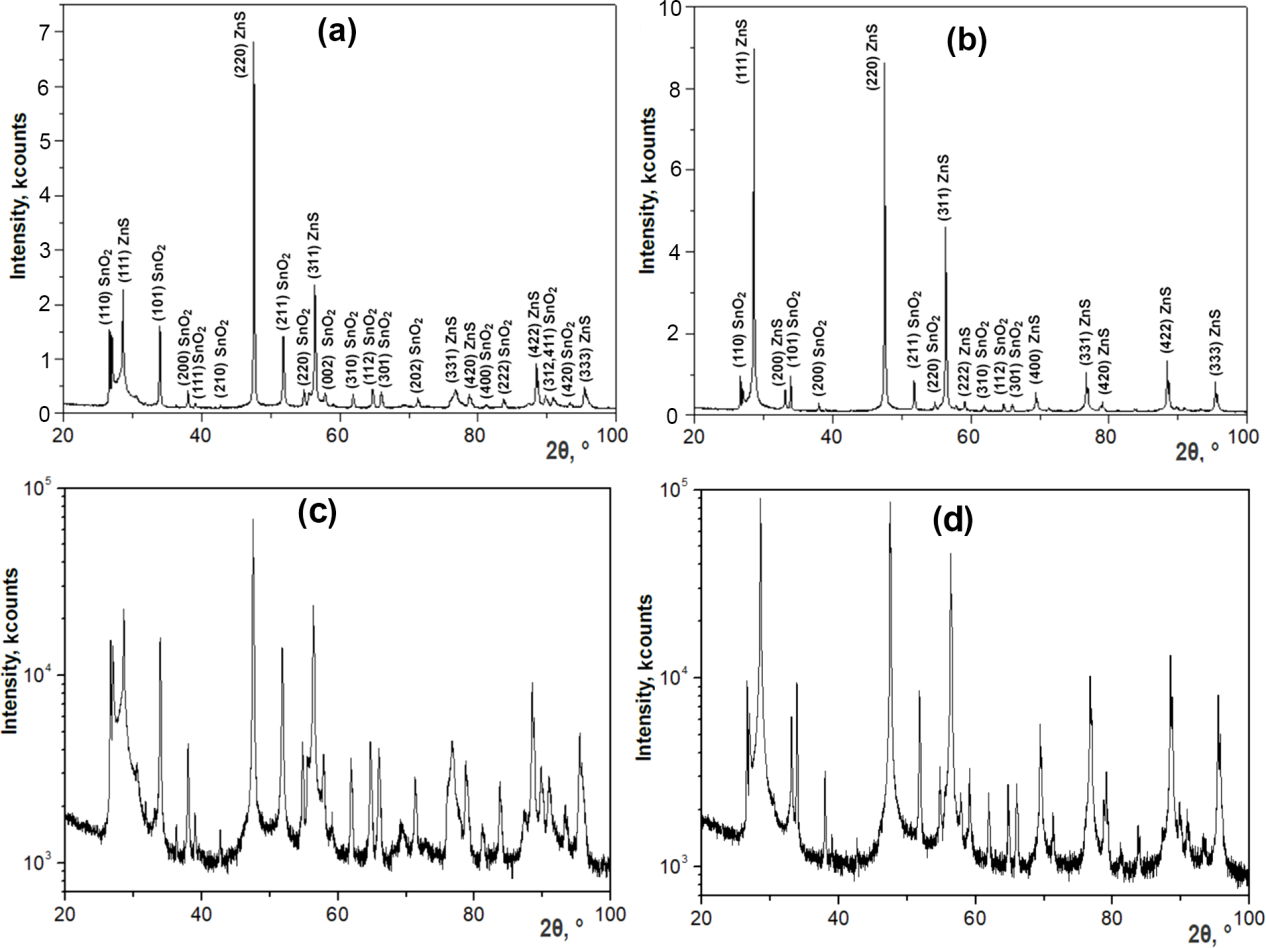
(a, c) XRD patterns of a ZnS aeromaterial sample produced in the 4 h procedure. (b, d) XRD pattern of a ZnS aeromaterial sample produced in the 8 h procedure. (a, b) are with linear intensity axes, while (c, d) are with logarithmic axes.

The XRD analysis of the sample produced in the 4 h procedure shows that it consists of two phases (Figure 2a). The reflexes are indexed to a cubic zinc blende ZnS phase according to the JCPDS cards no. 772100 and no. 05-0566 [29–30] and to a tetragonal rutile cassiterite SnO_2_ phase according to PDF 21-1250 and JCPDS card no. 41-1445 [31–32]. Whole powder pattern fitting (WPPF) weight fraction analysis revealed that the ZnS phase is 100% of zinc blende structure.

**Figure 3:**
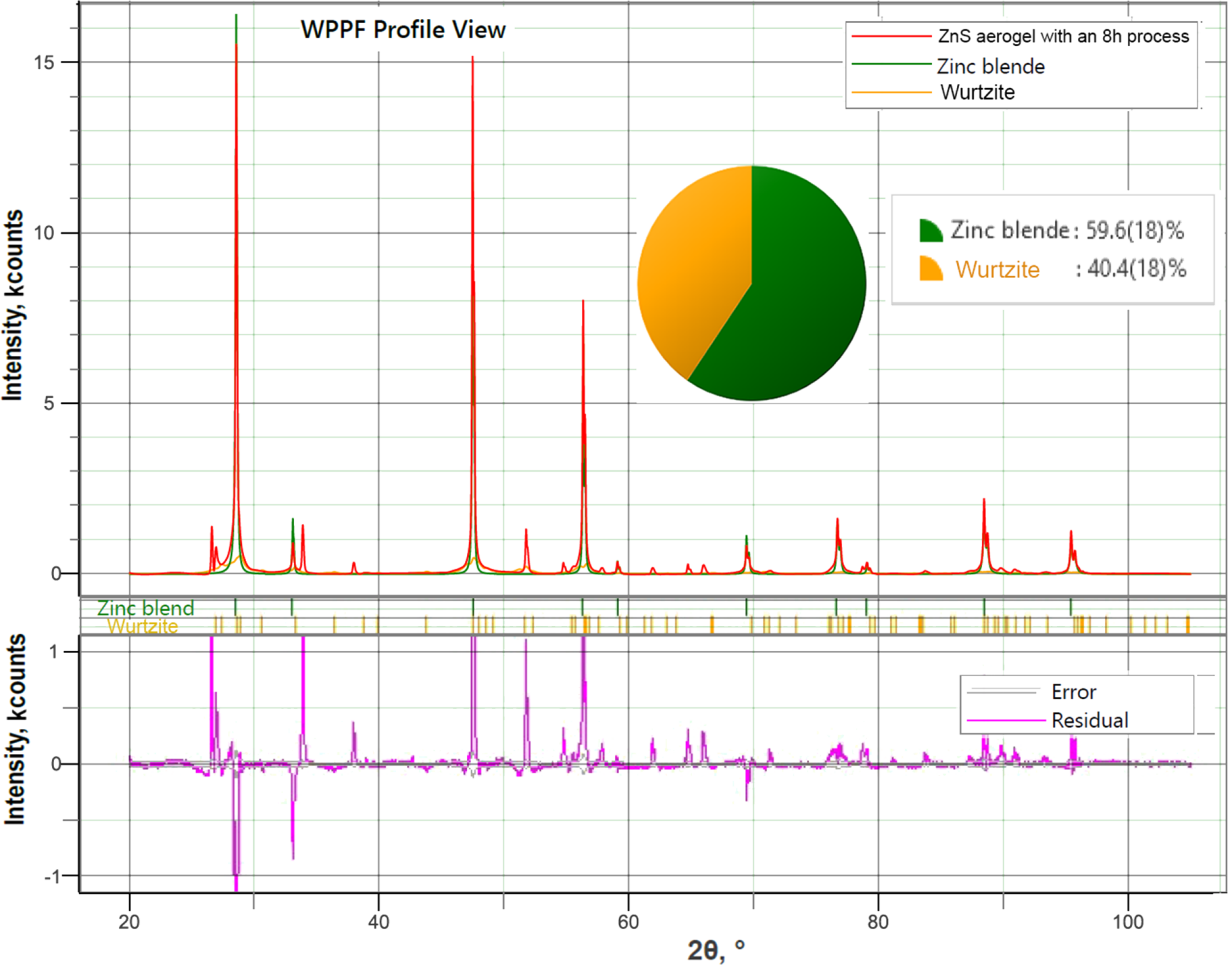
Results of the WPPF weight fraction analysis performed for the sample produced in the 8 h technological procedure.

The analysis of the XRD pattern in Figure 2b demonstrates that the SnO_2_ phase content in the sample produced in the 8 h procedure has decreased. At the same time, the WPPF weight fraction analysis performed for this sample (Figure 3) revealed that the ZnS component consists of two phases, namely 60% of a phase with cubic zinc blende structure and 40% of a phase with hexagonal wurtzite structure.

**Figure 4:**
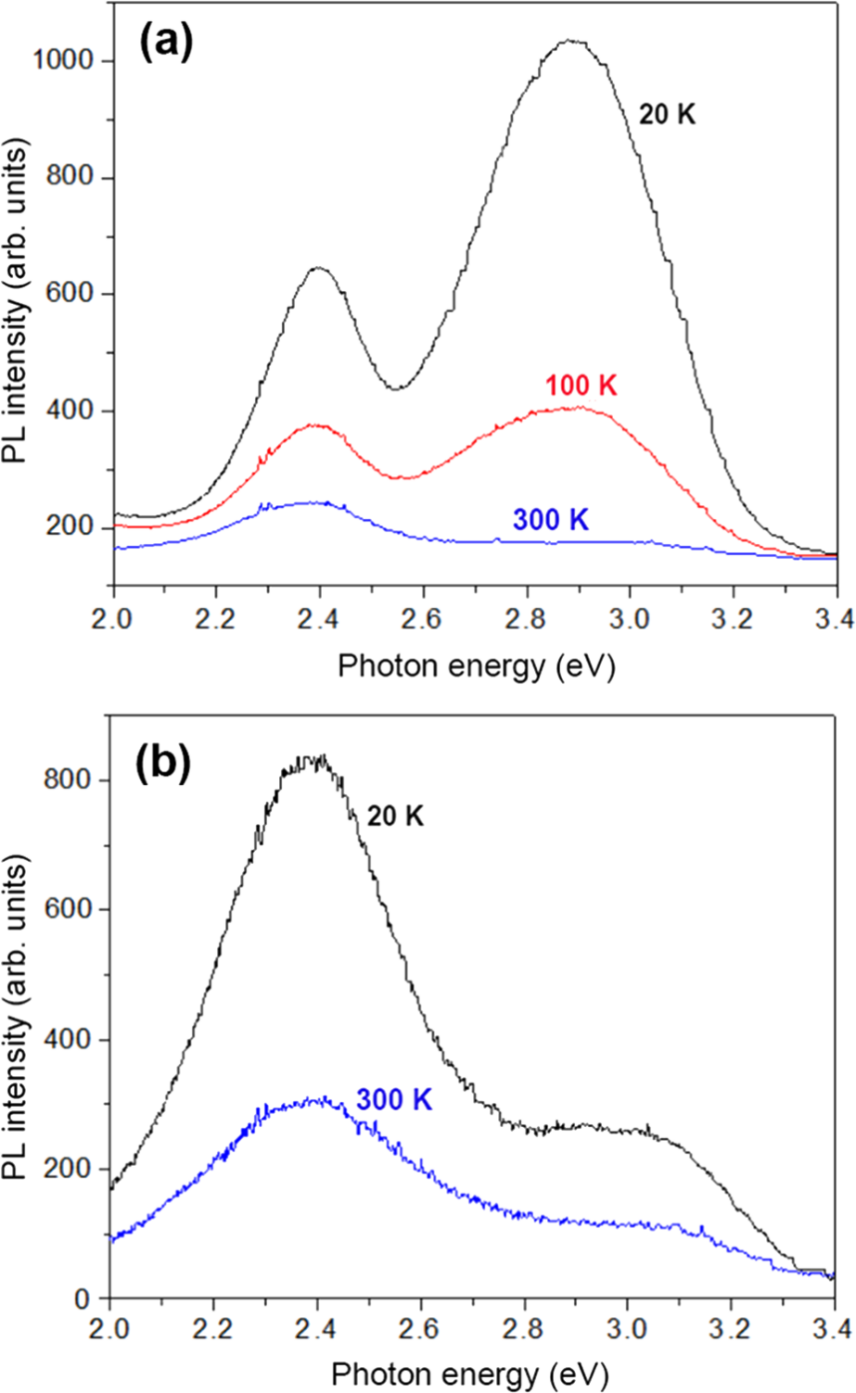
PL spectra of samples prepared in (a) the 4 h procedure and (b) the 8 h procedure measured in the deep level defect region at different temperatures under resonant excitation (325 nm laser line).

The good crystalline quality of the ZnS component of the aeromaterial was revealed by the XRD analysis. In order to assess the optical quality, PL spectra were analyzed under resonant near-bandgap and intraband excitation. The PL spectra of samples prepared in the technological procedures of 4 and 8 h were measured at temperatures from 300 to 20 K under excitation with the 325 nm laser line (3.81 eV), which corresponds to excitonic resonances in ZnS, see Figure 4. The spectra of both samples consist of two broad PL bands centered around 2.4 and 2.9–3.0 eV. However, the intensities of these bands are different. The position of these bands does not shift significantly with changing temperature. At room temperature, the intensity of the 2.4 eV band is higher than that of the 2.9–3.0 eV band in both samples. However, their behavior with decreasing temperature proves to be different. The intensity of both bands increases by a factor of around 3 with decreasing temperature from 300 to 20 K in the sample prepared in the 8 h procedure. In contrast, the intensity of both luminescence bands increases much faster in the sample prepared in the 4 h procedure, especially the intensity of the 2.9 eV band, which increases by more than one order of magnitude with decreasing the temperature from 300 to 20 K. Thus, the intensity of this PL band at low temperatures is higher than that of the 2.4 eV band in this sample. Apart from that, the position of the high-energy PL band is slightly shifted to higher energies in the sample prepared in the 8 h procedure, as compared with the sample prepared in the 4 h procedure. This could be due to the presence of the wurtzite ZnS phase in the sample prepared in the 8 h procedure, as indicated by the XRD analysis; it is known that the bandgap of the wurtzite ZnS phase is larger than that of the zinc blende phase.

**Figure 5:**
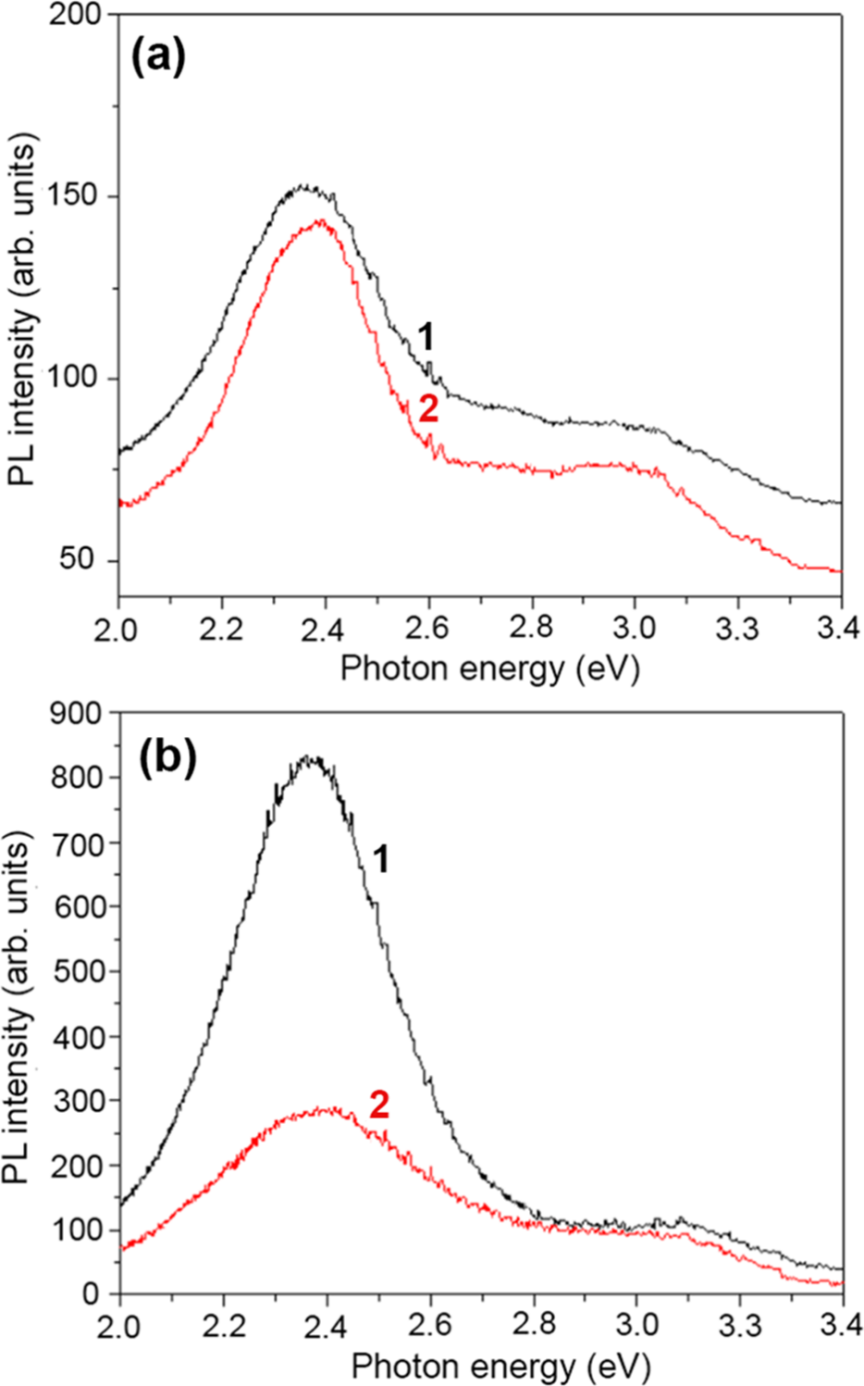
PL spectra of samples prepared in (a) the 4 h procedure and (b) the 8 h procedure measured in the deep level defect region at room temperature in air (curve 1) and in vacuum (curve 2) under resonant excitation (325 nm laser line).

The behavior of these PL bands is also different in the two samples when changing the environment, as deduced from Figure 5. The intensity of the PL bands in the sample prepared in the 4 h procedure is not significantly different when measured at room temperature in air and in vacuum (Figure 5a), while the intensity of the 2.4 eV band decreases significantly with changing the environment from air to vacuum in the sample prepared in the 8 h procedure (Figure 5b).

**Figure 6:**
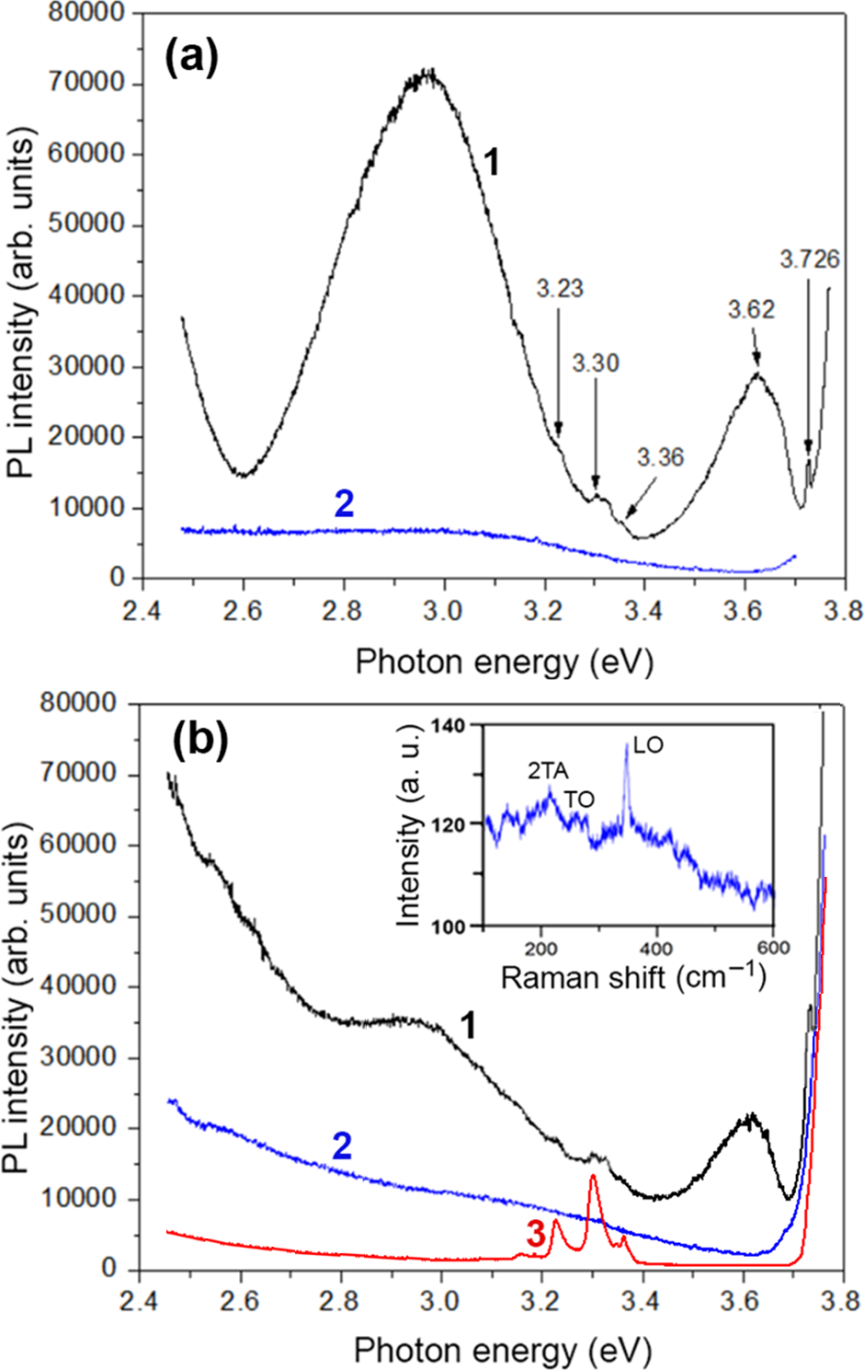
PL spectra of samples prepared in (a) the 4 h procedure and (b) the 8 h procedure measured in the near-bandgap region at 20 K (curve 1) and at room temperature (curve 2) under resonant excitation (325 nm laser line). The inset in (b) is the Raman scattering spectrum measured at room temperature. The spectrum measured at 20 K from the sacrificial ZnO template is presented for comparison (curve 3).

A PL band at 3.62 eV is observed in the spectra measured at low temperature in the near-bandgap region, as shown in Figure 6. Apart from that, a narrow emission peak is observed at 3.726 eV. This peak is assigned to multiphonon resonant Raman scattering (RRS) in ZnS since the quantum energy difference between the excitation laser line (3.814 eV) and the peak position (3.726 eV) is nearly equal to the 2LO phonon energy in ZnS. The non-resonant Raman scattering measured with the excitation by the 785 nm laser line, shown in the inset of Figure 6b, clearly indicates the presence of a Raman scattering peak at 350 cm^−1^ (43 meV), which corresponds to the LO phonon energy in both the zinc blende and the wurtzite ZnS phases. Apart from that, Raman scattering peaks are observed at 275–280 and 220 cm^−1^, which are assigned to the TO and 2LA Raman scattering, respectively [33–34]. Therefore, the quantum energy of the PL excitation laser line of 325 nm (3.814 eV) is in resonance with the exciton energy in zinc blende ZnS [35], and the conditions for RRS are satisfied [36], while the difference between the PL excitation laser line of 325 nm (3.814 eV) and the position of the 3.726 eV emission peak is nearly equal to the 2LO phonon energy (88 meV). The difference of a few inverse centimeters in the positions of phonon modes under resonant and non-resonant excitations could be related to several reasons such as (i) different contributions from the wurtzite and zinc blende phases in the samples, (ii) different contributions from LO modes with *A*_1_ and *E*_1_ symmetries in the ZnS wurtzite phase in non-polarized measurements, (iii) some contribution from the surface optical SO modes, which are inherent to porous and nanostructured materials [37–38], (iv) interaction of LO phonon modes with plasmons (LOPC), and (v) effects of strain and phonon confinement [37]. Nevertheless, both PL characterization and phonon mode analysis are indicative of a mixed-phase zinc blende/wurtzite composition of the prepared aero-ZnS material in concordance with the XRD analysis.

Apart from these major emission bands, a series of weaker features are observed in the range from 3.36 to 3.23 eV in the spectra of Figure 6 measured at low temperature. The features come from residual ZnO related to the sacrificial network of microtetrapods. To demonstrate this assumption, the PL spectrum measured for the initial ZnO template is presented in Figure 6b (curve 3). This spectrum consists of a weak PL band at 3.360 eV, related to the neutral donor D^0^X bound exciton emission in ZnO [39], and three higher-intensity PL bands at 3.30, 3.23, and 3.16 eV, associated with donor–acceptor (DA) recombination and its 1LO and 2LO phonon replicas, respectively [40–41].

The PL band in the near-bandgap region at 3.62 eV is related to a free-to-bound electron transition from a shallow donor to the valence band of ZnS [35]. The other two bands around 2.4 and 2.9–3.0 eV in the deep level defect region are the most common PL bands observed in various ZnS samples and have been associated with DA pair recombination [35]. Therefore, the photoluminescence properties of the prepared aero-ZnS materials, including the near-bandgap emission, are similar to those inherent to semiconductor ZnS single crystals.

**Figure 7:**
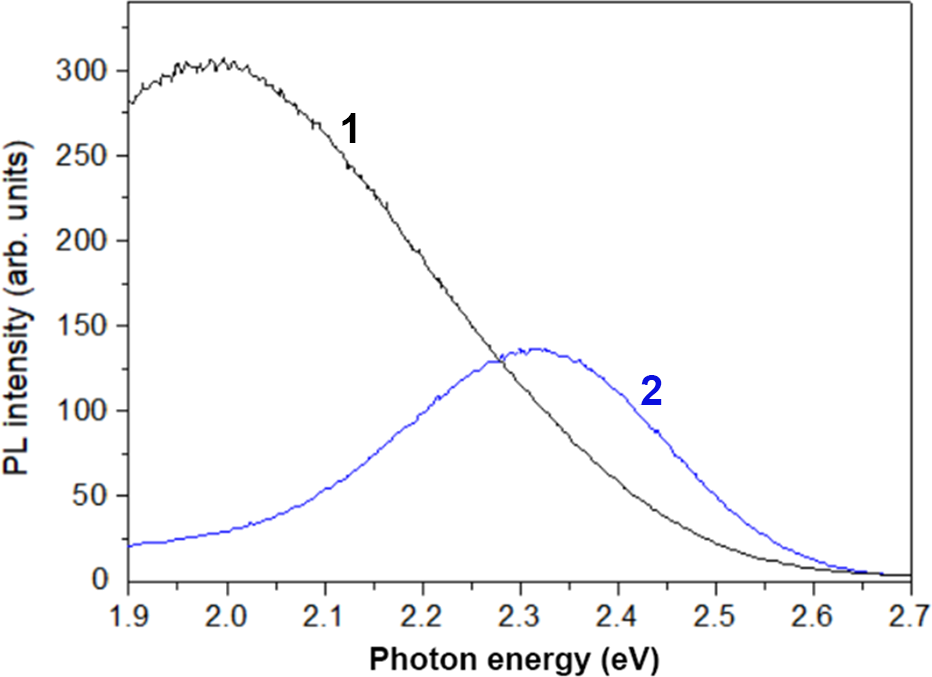
PL spectra of samples prepared in the 4 h procedure (curve 1) and the 8 h procedure (curve 2) measured at room temperature under intraband excitation (405 nm laser line).

The PL band around 2.4 eV is also excited by intraband-energy radiation with a wavelength of 405 nm (3.06 eV), as shown by curve 2 in Figure 7. However, under this excitation, the emission with longer wavelength around 625 nm (2.0 eV) dominates the emission spectrum of the sample prepared in the 4 h technological procedure. This emission may come from the SnO_2_ phase, which is present in this sample, as indicated by the XRD analysis discussed above.

**Figure 8:**
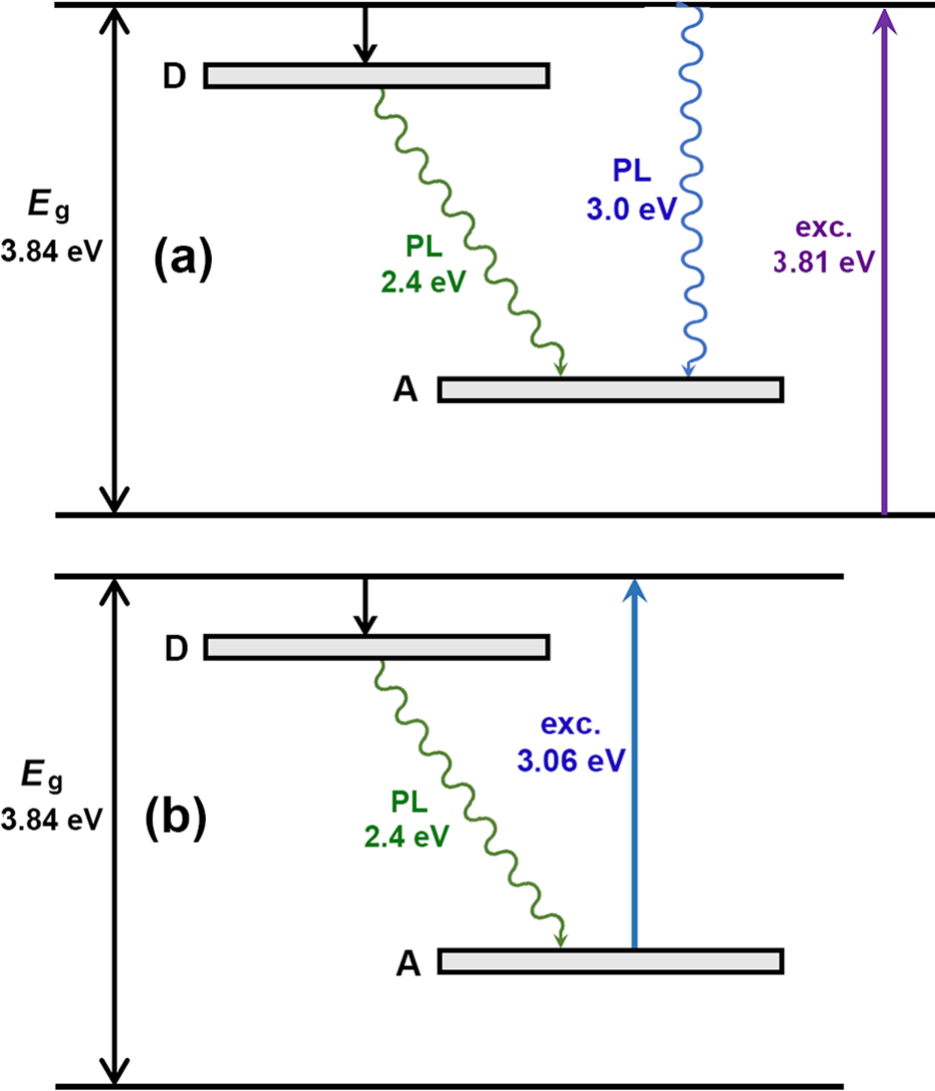
Schemes of deep energy levels and electronic transitions under (a) resonant and (b) intraband excitation.

A possible scheme of deep energy levels and electronic transitions accounting for the observed PL band in the deep level defect region for the ZnS component of the prepared aeromaterial is proposed in Figure 8. It includes a donor impurity band situated around 0.6 eV below the conduction band and an acceptor impurity band situated around 0.9 eV above the valence band.

Under resonant excitation by the 325 nm laser line, electron–hole pairs are created by intrinsic excitation, and the PL band at 2.9–3.0 eV is a result of free-to-bound transitions of electrons from the conduction band recombining with holes from the acceptor impurity band. The PL band at 2.4 eV is related to DA pair recombination of electrons from a donor impurity band with the holes from the acceptor impurity band, as shown in Figure 8a.

Under intraband excitation by the 405 nm laser line, the electrons from the acceptor impurity band are excited to the conduction band, and the PL band at 2.4 eV is again related to DA pair recombination of electrons from the donor impurity band with the holes from the acceptor impurity band, as shown in Figure 8b.

While the scheme of deep energy levels and electronic transitions in the prepared materials can be deduced with a high degree of reliability from the performed analysis, the determination of the microscopic origin of the recombination centers responsible for the observed emission bands is problematic with the available data. Nevertheless, some suppositions are possible. The fact that the intensity of the low-energy PL band around 2.4 eV decreases significantly with changing the environment from air to vacuum in the sample prepared in the 8 h technological procedure may indicate the participation of surface states in the recombination processes, including those related to oxygen species adsorbed by the huge surface of the aeromaterial, as previously observed in ZnS nanostructures [42]. Some of these states may be related to oxygen vacancies and complex centers. Under ambient conditions, some oxygen species from the air are adsorbed at the surface states, forming complexes with oxygen vacancies. These complexes may be responsible for the PL band around 2.4 eV. Under vacuum conditions, the oxygen species are desorbed from the surface, annihilating the formed complexes. As a result, the intensity of the PL band around 2.4 eV decreases in vacuum, as shown in Figure 5b.

As it has been shown previously, the oxygen vacancy defects in nanocomposite materials can act as active centers during photocatalytic oxidation processes by capturing photoinduced electrons, thus contributing to a substantial improvement of photocatalytic activity [43]. The adsorption of oxygen species from the environment on the sample surface promoted by oxygen vacancies results also in a strong interaction between the photoexcited electrons captured by oxygen vacancies and the adsorbed oxygen species.

The formation of the SnO_2_ phase in the prepared aero-ZnS materials revealed by XRD and PL spectral analyses (Figure 7) may be beneficial from the point of view of preparing various nanocomposites in a controlled manner, providing opportunities for bandgap engineering. This approach is interesting for the alignment of the conduction and valence bands of the nanocomposite components with respect to the HOMO and LUMO molecular orbitals of organic compounds subjected to photocatalytic degradation [18,44].

Specific photocatalytic properties of the aero-ZnS materials prepared by physical vapor transport have not been investigated in this paper. However, preliminary results suggest their suitability for the photocatalytic degradation of tetracycline. Nevertheless, focused investigations are needed concerning the optimization of the phase composition of the prepared materials for specific photocatalytic applications. Additional investigations of the surface states are also needed in view of the large specific surface area inherent to the produced aeromaterials and of the important role played by surface states in shifting the edge of the valence band, improving the visible-light photocatalytic properties, as shown by first-principle investigations in some nanomaterials [45]. Because of similarities in the morphology of aero-ZnS materials obtained by physical vapor transport to those produced by HVPE, one can expect that the materials prepared by physical vapor transport will also exhibit hydrophobic/hydrophilic properties suitable for microfluidic applications [16]. These experiments are ongoing in our laboratories, and their results will be published in a separate paper.

## Conclusion

The results of this study demonstrate a simple and cost-effective preparation of a ZnS aeromaterial with ZnO microtetrapod networks and Sn_2_S_3_ crystals as precursors. The composition and the crystallographic phase content of the aeromaterial can be controlled by the duration of the physical vapor transport of the Sn_2_S_3_ precursor and its chemical reaction with the ZnO microtetrapod networks. After short duration of the synthesis procedure, the produced aeromaterial is composed of a major zinc blende ZnS phase with some content of a tetragonal rutile SnO_2_ phase. The content of the SnO_2_ phase decreases with increasing the duration of the technological procedure with a concomitant transformation of the zinc blende phase to the wurtzite phase. The high crystalline quality of the phases is demonstrated by the narrow lines in the XRD pattern, while their good optical properties are indicated by the presence of the near-bandgap emission and the narrow lines of resonant Raman scattering. In spite of the good crystalline quality, emission bands related to donor and acceptor impurities are present in the photoluminescence spectra, including those associated with surface states. Taking into account that, according to previous reports, the surface states, including those related to oxygen species adsorbed at the aeromaterial surface, play an important role in photocatalytic properties, one may expect that the developed aero-ZnS material is a potential candidate for photocatalytic applications, as well as for sensor applications, as demonstrated for other aeromaterials. Apart from that, dual hydrophilic and hydrophobic properties demonstrated previously for aero-ZnS are prospective for microfluidic applications. One may expect that the physical vapor transport technology applied in this research will be suitable also for the preparation of other aero-semiconductor materials, increasing the number of potential applications.

## Experimental

The ZnS aeromaterial was prepared in evacuated quartz ampoules with an inner diameter of 15 mm, where Sn_2_S_3_ crystals as source material were placed at a distance of about 100 mm from the ZnO target consisting of a network of microtetrapods. The technological procedure was carried out in a two-zone horizontal furnace. The Sn_2_S_3_ crystals were sublimated at a temperature of 720 °C, while the temperature of ZnO microtetrapods was maintained at 690 °C. The vapors from the sublimated Sn_2_S_3_ crystals were physically transported to the ZnO microtetrapods. During the process of ZnS formation, the sacrificial ZnO was decomposed; consequently, a network of ZnS hollow microtetrapods was produced as a result of chemical reactions. The duration of the technological procedure was four and eight hours.

This technological installation is less sophisticated and, consequently, less expensive than the equipment for HVPE previously used for the preparation of aero-semiconductor materials [11–18], including aero-ZnS materials [16]. The HVPE installation consists of two chambers. In the first chamber, ZnS/ZnO core–shell structures are grown through transporting vapors from the CdS powder source to the ZnO substrate by a H_2_ flow. The final ZnS aero-semiconductor material is produced in the second chamber by heating the ZnS/ZnO core–shell structures in a H_2_ atmosphere. Thus, the sacrificial ZnO template is dissolved from the ZnS/ZnO core–shell structures. In contrast, in this paper, all technological procedures occur in a single quartz ampoule through physical vapor transport.

The sacrificial network of ZnO microtetrapods with an arm length of 10–30 µm was prepared at Kiel University in Germany by a simple flame transport approach, which is described in [46]. The produced powder was pressed in an inox chamber with dimensions of 10 × 10 mm^2^ to prepare 2 mm thick sample tablets. In order to increase the mechanical stability, the tablets with a density of 0.5 g/cm^3^ were annealed at 1000 °C for 1 h in air.

Morphology analysis was carried out with a VEGA TESCAN 5130 SEM instrument equipped with an EDX detector from Oxford Instruments.

The structural properties were investigated by X-ray diffraction measurements on a Rigaku MiniFlex 600 diffractometer with Cu Kα radiation (λ=1.540598 Å) in a standard 2θ Bragg–Brentano configuration, operating at 45 kV beam voltage and 15 mA beam current.

Photoluminescence (PL) was excited by the 325 nm line of a He–Cd laser, or by the 405 nm line of an Ondax LM-405-PLR-40-1 laser (30 mW) with the samples mounted on the cold station of a LTS-22-C-330 optical cryogenic system. The excitation with the 325 nm laser line is interesting from the point of view of ensuring resonant conditions of excitation since its photon quantum energy is in resonance with excitons in ZnS single crystals. The emission was analyzed in a quasi-backscattering geometry through a double SDL-1 spectrometer. The signal from a FEU-106 photomultiplier with SbKNaCs photocathode working in a photon counting mode was fed to a PC via a GPIB interface for further data processing.

Raman scattering (RS) spectra were recorded at room temperature with a Sol Instruments MS750 confocal system equipped with a laser excitation source of 785 nm (Coherent-Ondax LM series, optical power 7.5 mW). A 100× microscope objective lens was selected to focus the light on the sample surface. The system calibration was performed on a monocrystalline Si wafer with the main peak measured at 521 cm^−1^. A 1200 gr/mm grating with a resolution of 1 cm^−1^ was utilized.

## Data Availability

The data that supports the findings of this study is available from the corresponding author upon reasonable request.
